# Immunoinformatic Analysis of Calcium-Dependent Protein Kinase 7 (CDPK7) Showed Potential Targets for *Toxoplasma gondii* Vaccine

**DOI:** 10.1155/2021/9974509

**Published:** 2021-07-08

**Authors:** Ali Taghipour, Sanaz Tavakoli, Mohamad Sabaghan, Masoud Foroutan, Hamidreza Majidiani, Shahrzad Soltani, Milad Badri, Ali Dalir Ghaffari, Sheyda Soltani

**Affiliations:** ^1^Department of Parasitology, Faculty of Medical Sciences, Tarbiat Modares University, Tehran, Iran; ^2^Department of Parasitology and Mycology, School of Medicine, Isfahan University of Medical Sciences, Isfahan, Iran; ^3^Behbahan Faculty of Medical Sciences, Behbahan, Iran; ^4^USERN Office, Abadan University of Medical Sciences, Abadan, Iran; ^5^Zoonotic Diseases Research Center, Ilam University of Medical Sciences, Ilam, Iran; ^6^Medical Microbiology Research Center, Qazvin University of Medical Sciences, Qazvin, Iran

## Abstract

Apicomplexan parasites, including *Toxoplasma gondii* (*T. gondii*), express different types of calcium-dependent protein kinases (CDPKs), which perform a variety of functions, including attacking and exiting the host cells. In the current bioinformatics study, we have used several web servers to predict the basic features and specifications of the CDPK7 protein. The findings showed that CDPK7 protein has 2133 amino acid residues with an average molecular weight (MW) of 219085.79 D. The aliphatic index with 68.78 and grand average of hydropathicity (GRAVY) with -0.331 score were estimated. The outcomes of current research showed that the CDPK7 protein included 502 alpha-helix, 1311 random coils, and 320 extended strands with GOR4 method. Considering the Ramachandran plot, the favored region contains more than 92% of the amino acid residues. In addition, evaluation of antigenicity and allergenicity showed that CDPK7 protein has immunogenic and nonallergenic nature. The present research provides key data for more animal-model study on the CDPK7 protein to design an efficient vaccine against toxoplasmosis in the future.

## 1. Introduction

*Toxoplasma gondii* is a prevalent intracellular protozoan, which can infect a broad spectrum of mammals (i.e., human) and birds [1, 2]. Oocysts are the potential infective form in the life cycle of the parasite. Feline species as the only definitive hosts can contaminate the environment by shedding unsporulated oocysts through feces [3]. *T. gondii* is transferred by water/vegetables contaminated via mature oocysts and consumption of raw or semicooked meat from infected animals, vertical transmission from infected pregnant mothers to neonates, and blood transfusion [4–7]. Approximately one-third of human society has been exposed to *T. gondii*, worldwide [5, 8, 9]. Often *T. gondii* infection among immunocompetent people is asymptomatic or demonstrates mild symptoms, whereas in immunocompromised patients, it can cause a various range of clinical symptoms [6, 9, 10]. Toxoplasmosis in immunocompromised subjects can cause repeated attacks in the brain and manifests as encephalitis [11]. Moreover, toxoplasmosis in pregnant women can cause blindness, microcephaly, and mental retardation in the infant [6, 12]. Different factors, such as host's immune system status, genetic background, age, gender, contact with infected cats, environmental conditions, and diet and cultural habits, as well as the protozoan genotype, can affect the morbidity and mortality rate of *Toxoplasma* infection [13, 14].

Today, treatment of toxoplasmosis with conventional drugs can just limit the proliferation of tachyzoites at the beginning of infection, while these drugs cannot eradicate cystic forms of parasites in host tissue [15, 16]. In addition, taking these medications in pregnant women can have serious side effects, such as the possibility of teratogenic effects on the fetus [17]. Hence, the discovery and design of an effective vaccine to control and prevent toxoplasmosis is very important, especially in humans and domestic animals. In this regard, various *in silico*-based studies suggest various antigens as suitable candidates for vaccine design [18–32]. Calcium-dependent protein kinases (CDPKs) are a class of serine/threonine kinases that express in apicomplexans, ciliates, and plants [33]. In *T. gondii* as a member of the apicomplexan parasites, several CDPKs have been identified involving in critical functions in the different stages of the life cycle of parasite, including gliding motility (surface translocation), entry into (invasion), and exit from (egress) of host cells [34]. The CDPK7 is a crucial enzyme for division, growth, and maintenance of structural integrity of the *Toxoplasma* centrosome. As a result, TgCDPK7 knockdown is suggested as an important goal in achieving the right vaccine [35].

Computer-aided evaluation of different *T. gondii* proteins involved in various stages of life cycle can open new doors towards recognizing potent vaccine candidates through identification of highly immunogenic, nonallergenic, and nontoxic B- and T-cell epitopes [36]. Thereby, the present *in silico* study was performed to evaluate the crucial biochemical features and immunogenic epitopes of the CDPK7 protein by means of different bioinformatics servers.

## 2. Methods

### 2.1. CDPK7 Sequence

For this purpose, ToxoDB online website was used to obtain the whole amino acid sequence of *T. gondii* CDPK7 protein.

### 2.2. Physicochemical Characterization

We used the Expasy ProtParam online server to predict the physicochemical parameters of CDPK7 [37].

### 2.3. Prediction of Posttranslational Modification (PTM) Sites

The NetPhos 3.1 online tool was applied to predict phosphorylation location, and the CSS-Palm online server was applied to predict acylation location of the CDPK7 [38, 39].

### 2.4. Transmembrane Domains and Subcellular Location

The transmembrane regions and subcellular localization of *T. gondii* CDPK7 protein were assessed utilizing the TMHMM 2.0 and PSORT II web servers, respectively [38].

### 2.5. Secondary and Tertiary Structures

In this study, we employed the Garnier-Osguthorpe-Robson 4 (GOR4) online tool to forecast the secondary structure of CDPK7 protein [40]. Consequently, the three-dimensional (3D) model structures was used by SWISS-MODEL [38, 41].

### 2.6. The 3D Modeled Structure Refinement and Validation

GalaxyRefine was selected to develop and refine the quality of the template-based protein prediction [42]. To the Ramachandran plot validated the 3D structure of the protein, the SWISS-MODEL software was applied [43]. ProSA-web was used for evaluation of the whole quality of the model [44].

### 2.7. Linear and Conformational B-Cell Epitopes

We used a web-based Bcepred server to predict continuous B-cell epitopes exploiting physicochemical characteristics [45]. An online server of ABCpred was applied to predict B-cell epitopes [46]. Using the immune epitope database (IEDB), hydrophilicity [47], Bepipred linear epitope prediction [48], antigenicity [49], surface accessibility [50], beta-turn [51], and flexibility [52] were predicted. Afterwards, discontinuous B-cell epitopes were appraised by ElliPro [53] from the 3D structure of protein epitopes.

### 2.8. MHC-I and MHC-II Epitopes

To this aim, we used the IEDB website to evaluate the half-maximal inhibitory concentration (IC_50_) values of peptides that bind to the main histocompatibility complex (MHC) class I and class II molecules for CDPK7 [54, 55]. All predicted epitopes were then evaluated in terms of antigenicity using the VaxiJen v2.0 server.

### 2.9. Cytotoxic T-Lymphocyte (CTL) Epitopes

We applied CTLpred online website according to 75.8% accuracy [56]. Next, all predicted epitopes were evaluated regarding antigenicity using the VaxiJen v2.0 server.

### 2.10. Antigenic and Allergenic Profiles

The antigenicity of the full CDPK7 sequence was estimated by VaxiJen v2.0 [57]. The allergenic profile of CDPK7 was predicted by the AllergenFP v1.0 and AllerTOP v2.0 servers [58, 59].

## 3. Results

### 3.1. General Information of CDPK7

The amino acid structure of CDPK7 was obtained from the ToxoDB server with accession no. TGME49_228750. Based on the ProtParam database, the CDPK7 protein entails of 2133 amino acid residues with molecular weight of 219085.79 D, whereas theoretical pI was 5.79. The overall number of negatively (Asp+Glu) charged residues was 209, and positively (Arg+Lys) charged residues was 178. There are a total number of 30441 atoms. The half-life of the CDPK7 was predictable at 30 hours, >20 hours, and >10 hours for mammalian (*in vitro*), yeast (*in vivo*), and *Escherichia coli* (*in vivo*), respectively. In addition, the instability index of the CDPK7 protein presented an unstable nature with a value of 53.28. In addition, the aliphatic index was calculated 68.78, and GRAVY of the protein was estimated -0.331.

### 3.2. PTM Sites of CDPK7 Protein

**Figure 1:**
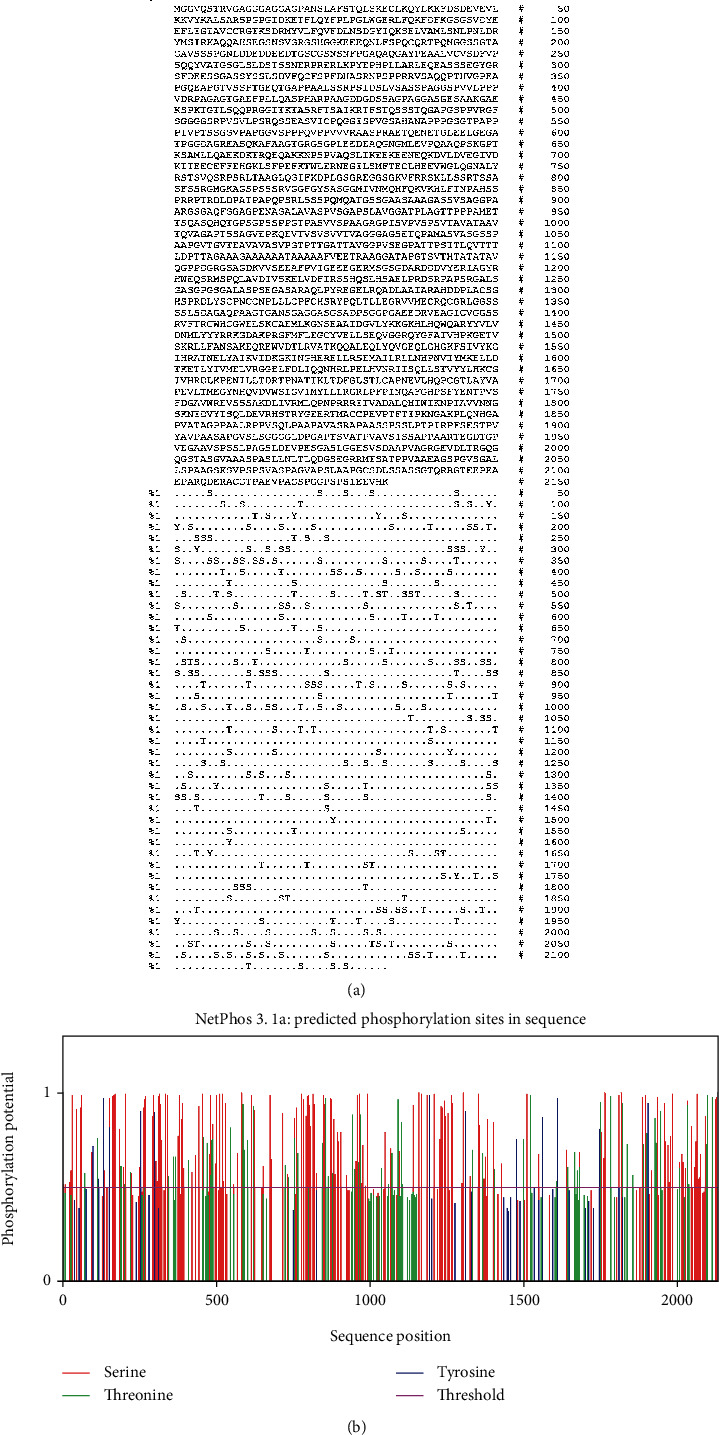
NetPhos server output for CDPK7 phosphorylation sites. (a) The number of predicted sites, based on S (serine), T (threonine), and Y (tyrosine); (b) prediction diagram of CDPK7 phosphorylation sites.

In the present research, the results exhibited that 269 phosphorylation sites (Thr: 64, Tyr: 13, and Ser: 192) (Figures 1(a) and 1(b)) and 30 acylation sites (Table 1) were recognized in the CDPK7, representing that the CDPK7 sequence is composed of 299 possible PTM sites.

### 3.3. Transmembrane Domains and Subcellular Location

**Figure 2:**
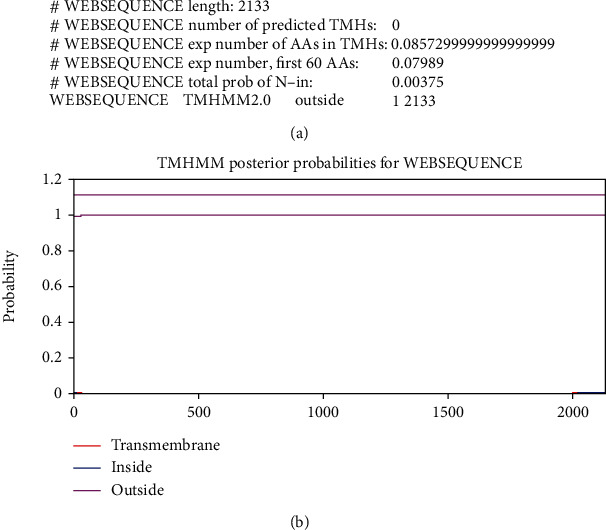
Transmembrane domains expected in CDPK7 protein. (a) Some statistics and a list of the location of the predicted transmembrane helices and the predicted location of the intervening loop regions. Length: the length of the protein sequence; number of predicted TMHs: the number of predicted transmembrane helices; Exp number of AAs in TMHs: the expected number of amino acids in transmembrane helices. If this number is larger than 18, it is very likely to be a transmembrane protein (or have a signal peptide); Exp number, first 60 AAs: the expected number of amino acids in transmembrane helices in the first 60 amino acids of the protein. If this number is more than a few, you should be warned that a predicted transmembrane helix in the N-term could be a signal peptide; total prob of N-in: the total probability that the N-term is on the cytoplasmic side of the membrane; (b) transmembrane domains expected in CDPK7 protein. (b) Analysis of the transmembrane domains of CDPK7.

Based on the TMHMM output, no transmembrane domain was found for CDPK7 (Figures 2(a) and 2(b)). Moreover, by PSORT II, the CDPK7 subcellular site was predicted as follows: 78.3% nuclear, 8.7% cytoplasmic, 8.7% plasma membrane, and 4.3% cytoskeletal.

### 3.4. Secondary and Tertiary Structures

**Figure 3:**
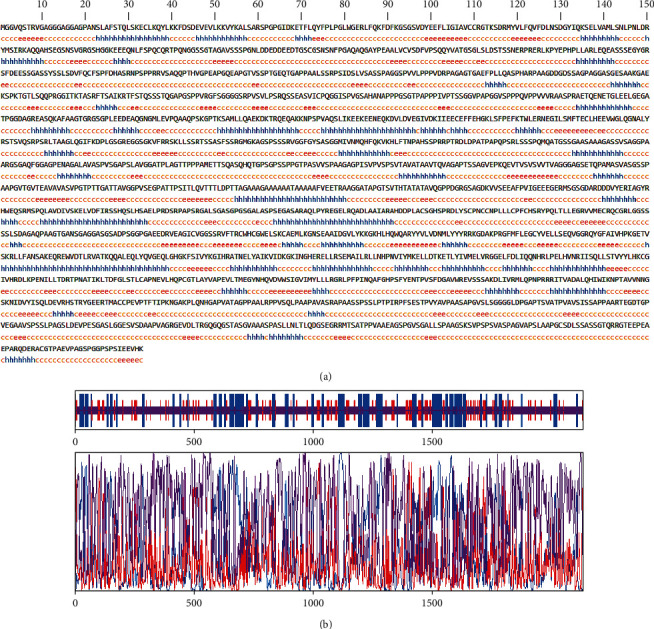
(a) GOR4 server results suggested that CDPK7 encompasses 502 alpha-helix, 320 extended strands, and 1311 random coils in secondary structure; (b) graphical result of the secondary structure prediction of CDPK7 using the GOR4 online server.

**Figure 4:**
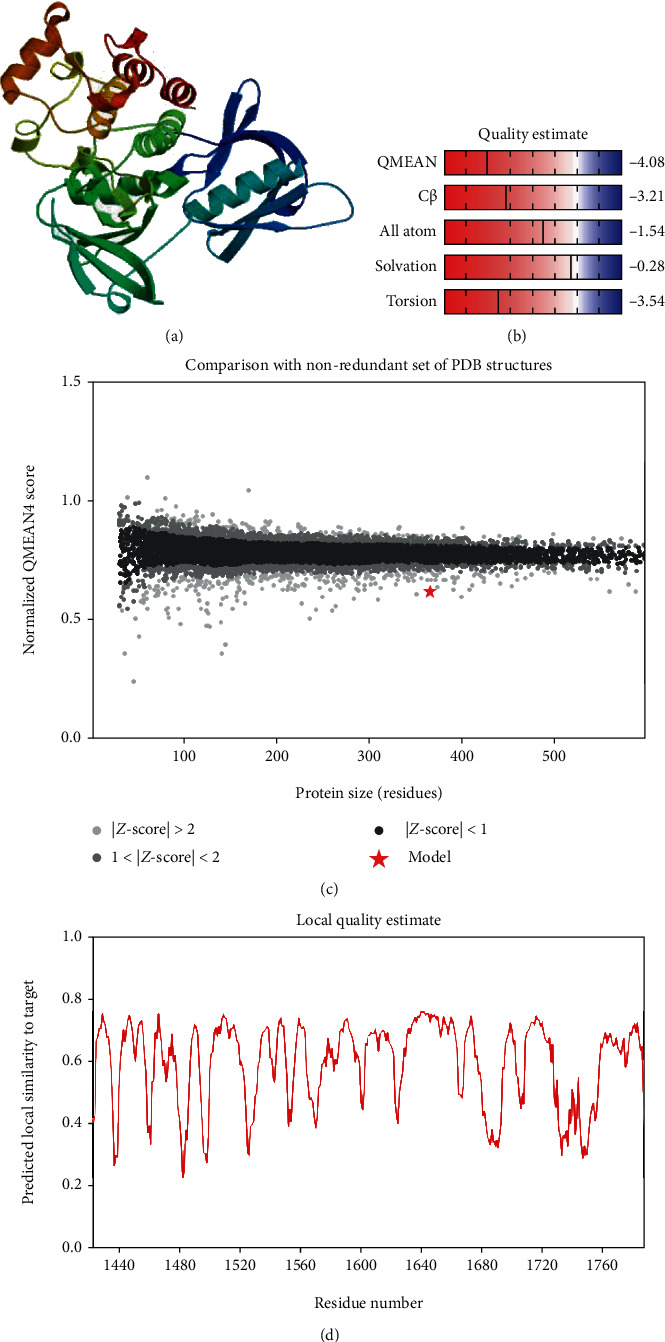
SWISS-MODEL server output. (a) Computed 3D model; (b) global quality estimate; (c) comparison with nonredundant set of PDB structures; (d) local quality estimate.

The secondary structure of CDPK7 was predicted via the GOR4 online server, suggesting 502 alpha-helix, 320 extended strands, and 1311 random coils (Figures 3(a) and 3(b)). Moreover, the SWISS-MODEL analysis is shown in Figures 4(a)–4(d).

### 3.5. Refinement and Validation of Tertiary Structure

**Figure 5:**
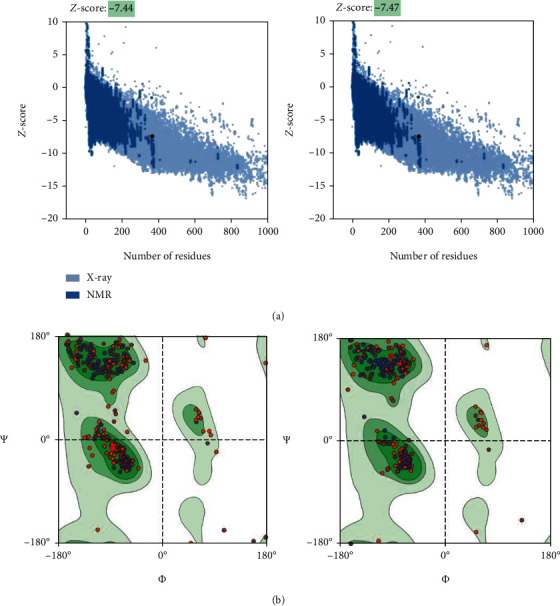
Validation of 3D model of CDPK7 protein. (a) The *Z*-score plot for 3D structure of predicted protein before and after refinement with ProSA-web server, respectively; (b) Ramachandran plot analysis of predicted structure.

Protein validation by means of the SWISS-MODEL server displayed that 92.86% of residues were situated in favored regions and 1.65% in the outlier regions. According to the Ramachandran plot, there were 97.80% residues in the favored region with 0.27% residues in the outlier regions of the refined model (Figure 5).

### 3.6. Predicted Linear and Discontinuous B-Cell Epitopes of the CDPK7 Protein

**Figure 6:**
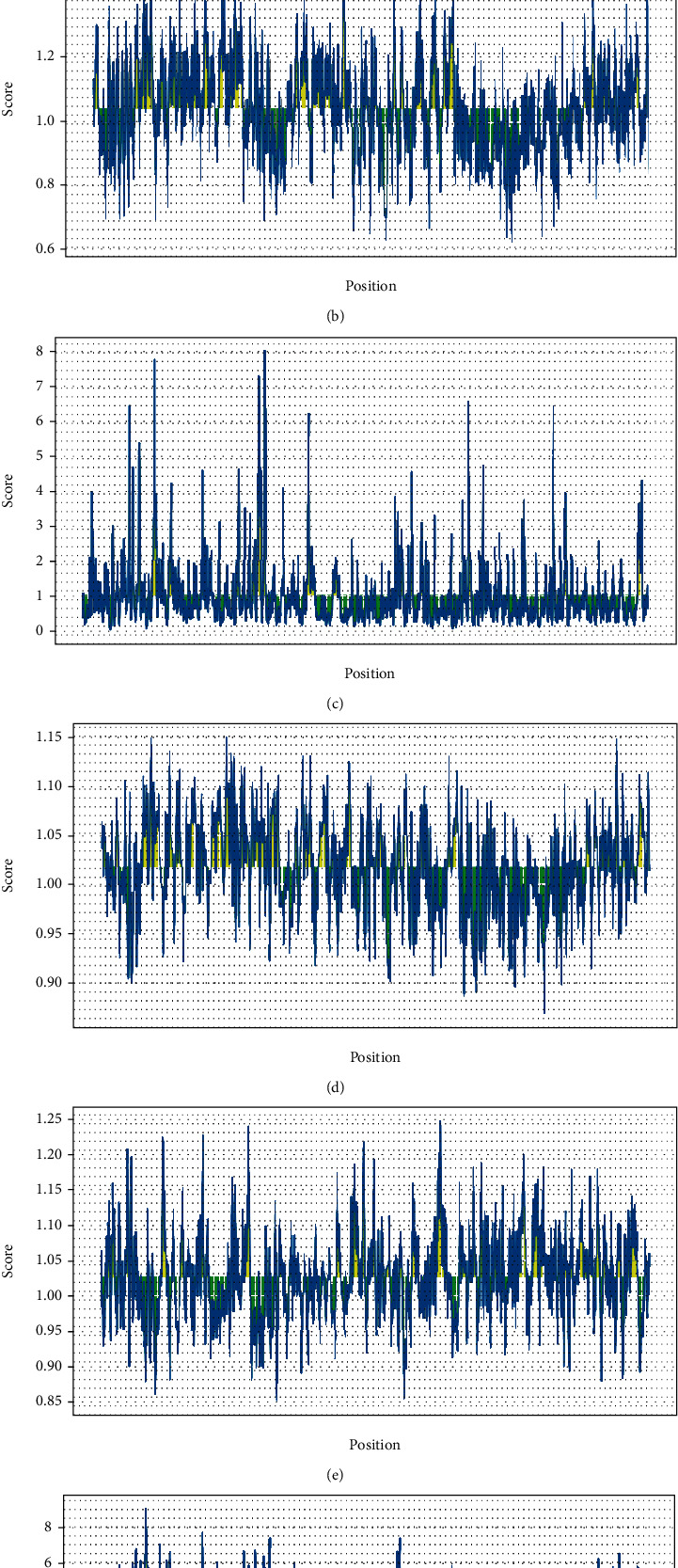
Propensity scale plots of CDPK7 protein. (a) Bepipred linear; (b) beta-turn; (c) surface accessibility; (d) flexibility; (e) antigenicity; (f) hydrophilicity. *x*-axis and *y*-axis represent position and score, respectively. The horizontal line indicates the threshold or the average score. Yellow colors (above the threshold) indicate favorable regions related to the properties of interest. Green color (below the threshold) indicates the unfavorable regions related to the properties of interest.

The predicted linear B-cell epitopes by the Bcepred are listed in Table 2. The outputs of the ABCpred server are tabulated in Table 3 (only the epitopes over scores of 0.75 are embedded in Table 3). The higher peptide score proposes the greater chance of being an epitope. The present server estimated 124 epitopes over 0.75 scores on the sequence, in which the linear epitope SSPPGTPASVVSPAAGAGPI (score: 0.95) had the greatest score. Four epitopes with over 0.95 scores were as follows: “SSPPGTPASVVSPAAGAGPI,” “EVPQAAQPSKGPTKSAMLLQ,” “GGVSPPPQVPPVVVRAASPR,” and “GETVSKRLLFANSAKEQREW.” The average score of antigenicity, beta-turn, flexibility, hydrophilicity, Bepipred linear epitope prediction, and surface accessibility for the CDPK7 protein using the IEDB online server was 1.026, 1.042, 1.017, 2.396, 0.350, and 1.00, respectively (Figure 6). Five discontinuous B-cell epitopes were predicted using the ElliPro server (Table 4).

### 3.7. MHC-Binding Epitopes

The results are listed in Tables 5 and 6. Epitopes were assessed regarding antigenicity, and those highly antigenic epitopes were finally selected.

### 3.8. CTL Epitope Prediction

The high-ranked CTL epitopes predicted by the CTLpred tool for CDPK7 protein are summarized in Table 7. Epitopes were assessed regarding antigenicity, and those highly antigenic epitopes were finally selected.

### 3.9. Antigenic and Allergenic Profiles

The antigenic profile of CDPK7 was conducted by the VaxiJen web server with score of 0.7074 (threshold: 0.5). Based on AllergenFP and AllerTOP v2.0 analyses, the CDPK7 protein was appraised as possible nonallergen.

## 4. Discussion

Toxoplasmosis is a significant menace to human society as well as livestock industry [2, 8, 60]. Thus, the design and improvement of an efficient vaccine against *T. gondii* infection is still a great challenge for researchers against toxoplasmosis in domestic animals and humans [61]. Recently, bioinformatics tools are more focused for rational vaccine design, with some advantage, including the following: (i) time- and cost-effectiveness; (ii) accurately targeting, long-lasting immunity with favorable polarity in cellular components; and (iii) elimination of undesired responses through specific, epitope-based construct design. Nevertheless, the obtained *in silico* results are only theoretical data which must be confirmed using wet lab experiments inevitably [62].

It has been known that CDPK7 contributes to several functions in *T. gondii* such as gliding movement, host-cell invasion, and egress as well as other vital growth processes [34]. Here, we conducted a comprehensive analysis of TgCDPK7, a member of the CDPK family in *T. gondii*. The amino acid sequence of CDPK7 comprises 2133 residues with an average MW of 219085.79 D, which characterizes a suitable antigenic nature (the peptides with MW more than 10 kDa are considered as good immunogens) [63]. According to the Expasy ProtParam server, GRAVY and the aliphatic index of the CDPK7 were achieved at -0.331 and 68.78, respectively. In summary, the great value of aliphatic index means that the peptide has more stability in a broad range of various temperatures. Moreover, the low/negative value of the GRAVY factor signifies the better interaction of peptide with the molecules of water. It is efficient to identify that PTMs have a fundamental role in cell stability [64]. The acquired outcomes show that CDPK7 comprises 299 potential PTM sites (269 phosphorylation and 30 acylation positions), representing that these positions may organize protein activity.

To predict the secondary structure of CDPK7, the GOR4 tool was recruited. The results of secondary structure of CDPK7 verified and included 502 (out of 2133) alpha-helix, 320 extended strands, and 1311 random coils. It is known that the key role of the proteins is related to their three-dimensional structure. As such, to comprehend the influences between both structures and functions, assessment of 3D structure is the key aim of expecting a protein's nature [65].

Humoral and cellular immunity are strongly stimulated in *T. gondii* infection [66, 67], in such a way that the establishment of IgG antibodies avoids the protozoan from attachment to the receptors of host cell [67]. Interferon-*γ* (IFN-*γ*), CD_4_^+^, and CD_8_^+^ T cells as the main members of T cells play a dynamic role in constraining acute and chronic infection. These major cytokines prevent the reactivation of bradyzoites in the host tissue cyst [66]. Epitope prediction has critical value to evaluate the specificity of antigen. Furthermore, epitope evaluation may reveal the pathogenesis and immune process of the pathogen in design vaccine researches [65, 68]. The strength of using *in silico* is the detection of the component epitopes that are critical for the interaction of antibodies and antigens. Several linear B-cell epitopes were predicted by the ABCpred server, among which those epitopes above 0.9 score were of great significance to be included in multiepitope vaccine constructs. Moreover, we applied the IEDB online server to evaluate the IC_50_ values of peptides that link to the MHC class I/II molecules for CDPK7. According to the obtained results from IEDB, the T-cell epitopes on CDPK7 have the capability to bind intensely to MHC class I and class II molecules. It is important to note that the lower IC_50_ values show the higher-level of affinity, which show an appropriate T-cell epitope.

Other the main stage, CTLpred is a special approach used to predict CTL epitopes, which is important in vaccine-related studies. This tool relies on elegant machine learning methods, such as ANN and SVM. We recognized the CTL epitopes using the CTLpred online database to select the top CDPK7 epitopes. The CTLpred server utilizes consensus and combined estimates, in line with these two methods [56]. Evaluation of antigenicity and allergenicity showed that CDPK7 protein has immunogenic and nonallergenic nature.

## 5. Conclusion

Well antigenicity, hydrophilicity, surface accessibility, and flexibility indexes were detected for CDPK7. Hence, we recommend that a suitable vaccine should be designed and verified both *in silico* and *in vivo* by the potential B- and T-cell epitopes predicted in this study.

## Figures and Tables

**Table 1:** The acylation sites of CDPK7 sequence.

ID	Position	Peptide	Score
TGME49_228750 CDPK7 (*T. gondii*)	34	STQLSKECLKQYLKK	1.129
TGME49_228750 CDPK7 (*T. gondii*)	109	FLIGIAVCCRGTKSD	1.996
TGME49_228750 CDPK7 (*T. gondii*)	110	LIGIAVCCRGTKSDR	5.494
TGME49_228750 CDPK7 (*T. gondii*)	187	QNLFSPQCQRTPQNG	0.526
TGME49_228750 CDPK7 (*T. gondii*)	222	DEEDTGSCGSNSNFP	5.293
TGME49_228750 CDPK7 (*T. gondii*)	244	YPEAALVCVSDFVPS	3.693
TGME49_228750 CDPK7 (*T. gondii*)	321	SLSDVFQCFSPFDHA	0.984
TGME49_228750 CDPK7 (*T. gondii*)	524	SSEASVICPQGGISP	2.536
TGME49_228750 CDPK7 (*T. gondii*)	706	VDKIIEECEFFEHGK	0.403
TGME49_228750 CDPK7 (*T. gondii*)	736	ILSMFTECLHEEVWG	1.821
TGME49_228750 CDPK7 (*T. gondii*)	1298	AHDDPLACSGHSPRD	5.591
TGME49_228750 CDPK7 (*T. gondii*)	1309	SPRDLYSCPNCCNPL	1.744
TGME49_228750 CDPK7 (*T. gondii*)	1312	DLYSCPNCCNPLLLC	8.015
TGME49_228750 CDPK7 (*T. gondii*)	1313	LYSCPNCCNPLLLCP	7.05
TGME49_228750 CDPK7 (*T. gondii*)	1319	CCNPLLLCPFCHSRY	2.719
TGME49_228750 CDPK7 (*T. gondii*)	1322	PLLLCPFCHSRYPQL	2.865
TGME49_228750 CDPK7 (*T. gondii*)	1340	EGRVVMECRQCGRLG	2.295
TGME49_228750 CDPK7 (*T. gondii*)	1343	VVMECRQCGRLGGSS	2.929
TGME49_228750 CDPK7 (*T. gondii*)	1395	DRVEAGICVGGSSRV	5.406
TGME49_228750 CDPK7 (*T. gondii*)	1406	SSRVFTRCWHCGWEL	0.108
TGME49_228750 CDPK7 (*T. gondii*)	1409	VFTRCWHCGWELSKC	1.424
TGME49_228750 CDPK7 (*T. gondii*)	1416	CGWELSKCAEMLKGN	4.272
TGME49_228750 CDPK7 (*T. gondii*)	1474	GFMFLEGCYVELLSE	1.626
TGME49_228750 CDPK7 (*T. gondii*)	1649	TVYYLHKCGIVHRDL	1.164
TGME49_228750 CDPK7 (*T. gondii*)	1683	DFGLSTLCAPNEVLH	1.6
TGME49_228750 CDPK7 (*T. gondii*)	1693	NEVLHQPCGTLAYVA	1.927
TGME49_228750 CDPK7 (*T. gondii*)	1828	GEERTMACCPEVPTF	4.139
TGME49_228750 CDPK7 (*T. gondii*)	1829	EERTMACCPEVPTFT	7.362
TGME49_228750 CDPK7 (*T. gondii*)	2080	PSLAAPGCSDLSSAS	3.862
TGME49_228750 CDPK7 (*T. gondii*)	2110	ARQDERACGTPAEVP	6.173

**Table 2:** Epitopes predicted in CDPK7 protein by different parameters based on the Bcepred online server.

Prediction parameter	Epitope sequence
Hydrophilicity	GAGGGAGGAG; KKFDSDEVEV; KGSGSVDYEE; CRGTKSDRM; AQQAHSEGSNSVGRGSHGGKEEEQNL; SPQCQRTPQNGGSSGTAGA; SPGNLDDEDDEEDTGSCGSNSN; SLDSTSSNERPRER; EQEASSSEGYGRSFDEESSGASSYSS; DHASRNP; GPEAPGQEAPGT; SSPTGEQTGAP; SASSPAGGS; DRPAGAGTGAE; RPAAGDDGDSSAGPAGGASGESAAKGAEKSPKTGT; SQQPRGG; STQSSSTQGAPGS; SGGGGSRP; PSRQSSEASV; SPRAETQENETGLEE; GEGATPGGDAGREASQKA; AGTGRGSGPLEEDEAQGNG; QPSKGPTKSA; QAEKDKTRQEQAKKNPS; IKEEKEENEQKDV; GSGREGGSGKV; SSRTSSAS; GKAGSPSSSRVGG; TNPAHSSPRRPTRD; QATGSSGAASA; ARGSGAQ; GGAGPENAGA; ETTSQASQHQTGPSGPSSPP; GVEPKQE; AGGGAGSETQPA; ASGSSPAA; SEGPATT; DPTTAGA; EETRAAGG; QGPPDGRGSAGDKV; IGEEEGERMSGSGDARDDDVYER; DSRPAPS; SGASGPGSGA; ASPSEGASAR; ARAHDDP; SGHSPRD; SSSSLSDAGAQP; AGTGANSGAGGASGSADPSGGPGAEEDRVEAG; KGNSEAA; RRKGDAKPRG; SEQVGGRQ; KGETVSKR; ANSAKEQRE; DKGKING; TDRTPNAT; EVSSSAKD; VNNGSKNID; DEVRHSTRYGEERT; AASSPSS; DPGAPTS; AARTEGDTGPVEG; DEVPESG; GGESVSDAA; AGRGEVD; TRGQGQGSTASG; TLQDGSEGRR; AEAGSPG; SSASSGTQRRGTEEPEAEPARQDERACGT; GSPGGPS
Flexibility	LAFSTQL; QYLKKFD; KALSARSPG; QKFDFKGSGS; AVCCRGTKSD; QQAHSEGSNSVGRGSHGGKEEE; QCQRTPQNGGSSGTAGAVSSSPGNLDDEDDEEDTGSCGSNS; SGLSLDSTSSNERPR; ARLEQEASSSE; RSFDEESSGA; FDHASRNPSPP; GTVSSPTGEQ; PAALSSRP; VSASSPAG; PAAGDDGDSS; GPAGGASG; SAAKGAEKSPKTGTLSQQPRGGITKTA; SAIKRTFSTQSSSTQ; PPVRGFSGGGGSRP; SVLPSRQSSE; NAPPPGSG; PIVPTSSG; PRAETQENE; GDAGREAS; FAAGTGRGS; QAAQPSKGPT; LQAEKDKTRQEQAKKNP; SLIKEEKEENEQ; ALYRSTSVQSRPS; KDPLGSGREGGSG; SKLLSSRTSSASFSSRGMGKAGSPSSSRV; NPAHSSPRRPT; APQPSRLSSSPQMQATGSS; PAARGSG; SQHQTGPSGPSSPP; TVAGGGAGSET; ASVASGSS; AVQGPPDGRGSAG; PVIGEEEGERMSGSGDA; RHWEQSR; DFIRSSH; AELPRDSR; GALSGASGPG; LACSGHSP; RQCGRLGGSSSSL; TGANSGAGGASGSADPSGG; GICVGGSS; AEMLKGNS; YYYRRKGD; VHPKGETVS; LFANSAKEQ; GKINGHE; ILLTDRT; AVWREVSSSA; RMLQPNPR; TAVVNNGS; APAASSPS; TPIRPFS; PGVSLSG; PAARTEGD; SGASLGGESV; VDLTRGQGQGSTA; LTLQDGSEGRRM; PAAGSKSV; SDLSSASSGTQRRGTE; VPAGSPGGPS
Accessibility	STQLSKECLKQYLKKFDSDEV; VLKKVYKAL; PGIDKETFLQY; GERLFQKFDFKG; CRGTKSDRMYV; SDGYIQKSEL; NLPNLDRYMSIRKAQQAHSEG; SHGGKEEEQNLFSPQCQRTPQNGG; PGNLDDEDDEEDTGS; DSTSSNERPRERLKPYEPHPL; ARLEQEASSSEGYGRSFDEESS; DHASRNPSPPRRVSAQQPTH; PEAPGQE; SSPTGEQTGAPP; PPPVDRPA; QASPHAR; AKGAEKSPKTGTLSQQPRGGITKTASRFTSAIKRTFSTQSSSTQG; LPSRQSSEAS; SPPPQVPP; ASPRAETQENETGLEE; GREASQKA; GPLEEDEAQGN; PQAAQPSKGPTKSA; LQAEKDKTRQEQAKKNPSPVAQSLIKEEKEENEQKDVLD; FKTWLERNEG; YRSTSVQSRPSRLTA; REGGSGKVFRRSKLLS; GSPSSSR; NMQHFQKVKH; PAHSSPRRPTRDLDPATPAPQPSRLSSSPQMQ; ETTSQASQHQTGPS; GVEPKQEVTV; EETRAAG; QGPPDGRGSAGDKVVSEE; GEEEGERMS; SGDARDDDVYERIAGYRHWEQSRMSPQ; RSSHQSL; SAELPRDSRPAPSRG; RAQLPYREGELRQAD; ARAHDDPL; SGHSPRDLYS; CHSRYPQLTL; PGAEEDRVEA; VLYKKGKHLHQWQARYY; NMLYYYRRKGDAKPRGF; EQVGGRQ; KGETVSKRL; ANSAKEQREWVDT; RVATKQQALEQ; IHRATNELY; KVIDKGKINGHERELLRSE; RLLNHPN; KELLDTKETLY; LIQQNHRLPEL; VHRDLKPENI; LTDRTPNAT; MEGYNHQ; HPSFYENTPVS; RMLQPNPRRRITV; VNNGSKNID; SQLDEVRHSTRYGEERTMA; IPKNGAKPLQNHG; RPFSEST; PPAARTEGDTG; QDGSEGRRMTSAT; SKSVPSPS; SSGTQRRGTEEPEAEPARQDERAC; PSPSIEE
Turns	SCGSNSNFPG; DSTSSNE; YSCPNCCNPL; LLNHPNVI
Exposed surface	KECLKQYLKKFDSDE; VLKKVYKAL; RGTKSDR; GKEEEQNL; DDEDDEEDT; SSNERPRERLKPYEPHP; RNPSPPRR; AEKSPKTG; LQAEKDKTRQEQAKKNPSPV; SLIKEEKEENEQKDV; KVFRRSK; QHFQKVK; SPRRPTRDL; EPKQEVT; VLYKKGKHLH; LYYYRRKGDAKPR; NSAKEQREWV; KQQALEQ; KVIDKGK; KELLDTK; HRDLKPEN; LQPNPRRRIT; QRRGTEE; EPARQDER; EEVHK
Polarity	LSKECLKQYLKKFDSDEVEVLKK; GERLFQKF; RGTKSDR; RYMSIRKAQQAH; RGSHGGKEEEQNLF; GNLDDEDDEEDTGS; SSNERPRERLKPYEPHP; LARLEQEAS; GRSFDEES; AEKSPKTG; PRAETQENETGLEE; GREASQKA; GPLEEDEAQG; LQAEKDKTRQEQAKKNP; QSLIKEEKEENEQKDVLDVE; VDKIIEECEFFEHGKLSF; EFKTWLERNEGIL; FTECLHEEVWGL; REGGSGKVFRRSKLLS; QHFQKVKHLFT; AHSSPRRPTRDLD; GVEPKQEVT; AFVEETRAAG; DKVVSEE; IGEEEGERMSGSGDARDDDVYERIAGYRHWEQSRM; HSAELPRDSR; LPYREGELRQA; IARAHDDPL; EGRVVMECRQCGR; PGAEEDRVEAG; ELSKCAE; LYKKGKHLHQWQ; LYYYRRKGDAKPR; IVHPKGETVSKRL; NSAKEQREWVD; EQLGHGK; VYKGIHRATNEL; KVIDKGKINGHERELLRSEM; KELLDTKE; ELVRGGE; QNHRLPELHVNRI; HKCGIVHRDLKPEN; QPNPRRRITVA; QLDEVRHSTRYGEERTMAC; DGSEGRRMTS; TQRRGTEEPEAEPARQDERAC; PSIEEVHK
Antigenic propensity	QLSKECLKQYLK; VEVLKKVYK; FLQYFPLPGL; VCCRGTK; MYVLFQVFDL; NLFSPQCQ; LVCVSDFVPSQQYV; YEPHPLL; YSSLSDVFQCFSPFDH; PSIDSLVS; GGSPVVLPPPVD; SRPVSVLPSRQS; SVICPQGG; PPPIVPTS; VSPPPQVPPVVVR; QKDVLDVEGIV; ECLHEEVW; FQKVKHLF; GPISVPVSPSVT; QEVTVSVSVVTV; PSITLQVTTTL; IVSKELVDFIRS; PRDLYSCPNCCNPLLLCPFCHSRYPQLTLLEGRVVMECRQCGRL; ICVGGSS; VFTRCWHC; IDGVLYK; RYYVLVDNML; FLEGCYVELLSEQVG; TVSKRLLF; LEQLYQV; GKFSIVYKGIH; ILRLLNHPNV; KETLYIVMELVR; LFDLIQQ; RLPELHVNRIISQLLSTVYYLHKCGIVHRD; FGLSTLC; EVLHQPCGTL; YNHQVDVWSIGVIMYLLLRGRL; LIVRMLQ; IDVYISQLD; CCPEVPTF; LRPPVSQLP; VSPSSLP; SLLNLTLQ; SVPSPSV

**Table 3:** The predicted B-cell epitopes via the ABCpred tool.

Rank	Sequence	Start position	Score
1	SSPPGTPASVVSPAAGAGPI	965	0.95
1	EVPQAAQPSKGPTKSAMLLQ	638	0.95
1	GGVSPPPQVPPVVVRAASPR	564	0.95
1	GETVSKRLLFANSAKEQREW	1497	0.95
2	DVLDVEGIVDKIIEECEFFE	691	0.94
2	EEDEAQGNGMLEVPQAAQPS	627	0.94
2	GAPTSVATPVAVSISSAPPA	1922	0.94
3	KNGAKPLQNHGAPVATAGPP	1839	0.93
4	ALEQLYQVGEQLGHGKFSIV	1528	0.92
5	GAPSLAVGGATPLAGTTPPP	927	0.91
5	FGYSASGGMIVNMQHFQKVK	821	0.91
5	KDKTRQEQAKKNPSPVAQSL	660	0.91
5	EDTGSCGSNSNFPGAQAQGA	217	0.91
5	LPAAPAVASRAPAASSPSSL	1868	0.91
5	DVWSIGVIMYLLLRGRLPFP	1714	0.91
5	VYKGIHRATNELYAIKVIDK	1547	0.91
5	SAKEQREWVDTLRVATKQQA	1509	0.91
6	TPVYAVPAASAPGVSLSGGG	1898	0.90
6	LIQQNHRLPELHVNRIISQL	1620	0.90
6	SGDARDDDVYERIAGYRHWE	1184	0.90
6	VAGAPTSSAGVEPKQEVTVS	1003	0.90
7	QATGSSGAASAAAGASSVSA	877	0.89
7	RSKLLSSRTSSASFSSRGMG	789	0.89
7	VGSAHANAPPPGSGTPAPPP	532	0.89
7	LYYYRRKGDAKPRGFMFLEG	1454	0.89
8	AGALAVASPVSGAPSLAVGG	916	0.88
8	PAAGDDGDSSAGPAGGASGE	424	0.88
8	SEAAIDGVLYKKGKHLHQWQ	1424	0.88
9	KNPSPVAQSLIKEEKEENEQ	670	0.87
9	GGDAGREASQKAFAAGTGRG	603	0.87
9	SAGPAGGASGESAAKGAEKS	433	0.87
9	ASRNPSPPRRVSAQQPTHVG	328	0.87
9	PHPLLARLEQEASSSEGYGR	281	0.87
9	SSNERPRERLKPYEPHPLLA	267	0.87
9	TPAEVPAGSPGGPSPSIEEV	2112	0.87
9	SSLSDAGAQPAAGTGANSGA	1351	0.87
9	KQEVTVSVSVVTVAGGGAGS	1016	0.87
10	STSVQSRPSRLTAAGLQGIF	752	0.86
10	LIKEEKEENEQKDVLDVEGI	679	0.86
10	LQASPHARPAAGDDGDSSAG	416	0.86
10	AQAQGAYPEAALVCVSDFVP	231	0.86
10	GEERTMACCPEVPTFTIPKN	1821	0.86
10	RCWHCGWELSKCAEMLKGNS	1405	0.86
10	MSPQLAVDIVSKELVDFIRS	1207	0.86
10	VASGSSPAAPGVTGVTEAVA	1044	0.86
11	TQGAPGSPPVRGFSGGGGSR	488	0.85
11	KTASRFTSAIKRTFSTQSSS	468	0.85
11	EESSGASSYSSLSDVFQCFS	304	0.85
11	GSPGVSGALLSPAAGSKSVP	2042	0.85
11	GDKVVSEEAFPVIGEEEGER	1161	0.85
12	ISVPVSPSVTAVATAAVTQV	984	0.84
12	NMQHFQKVKHLFTNPAHSSP	832	0.84
12	ASQKAFAAGTGRGSGPLEED	610	0.84
12	VEETRAAGGATAPGTSVTHT	1125	0.84
12	TVAGGGAGSETQPAMASVAS	1027	0.84
13	DKIIEECEFFEHGKLSFPEF	700	0.83
13	PGIDKETFLQYFPLPGLWGE	64	0.83
13	GYRHWEQSRMSPQLAVDIVS	1198	0.83
13	TTGATTAVGGPVSEGPATTP	1072	0.83
14	QTGPSGPSSPPGTPASVVSP	958	0.82
14	GTLSQQPRGGITKTASRFTS	456	0.82
14	AGAVSSSPGNLDDEDDEEDT	200	0.82
14	PCGTLAYVAPEVLTMEGYNH	1692	0.82
14	NSVGRGSHGGKEEEQNLFSP	166	0.82
14	LLSTVYYLHKCGIVHRDLKP	1639	0.82
14	GFAIVHPKGETVSKRLLFAN	1489	0.82
14	GFMFLEGCYVELLSEQVGGR	1467	0.82
14	CRQCGRLGGSSSSLSDAGAQ	1340	0.82
14	LSGASGPGSGALASPSEGAS	1249	0.82
14	KELVDFIRSSHQSLHSAELP	1218	0.82
14	ATAAAAAFVEETRAAGGATA	1117	0.82
14	AGAAAAAATAAAAAFVEETR	1110	0.82
15	DPATPAPQPSRLSSSPQMQA	859	0.81
15	FFEHGKLSFPEFKTWLERNE	708	0.81
15	TGLEELGEGATPGGDAGREA	591	0.81
15	APPAALSSRPSIDSLVSASS	369	0.81
15	SPTGEQTGAPPAALSSRPSI	361	0.81
15	EEPEAEPARQDERACGTPAE	2096	0.81
15	PAARTEGDTGPVEGAAVSPS	1940	0.81
15	KAQQAHSEGSNSVGRGSHGG	156	0.81
15	VGGPVSEGPATTPSITLQVT	1079	0.81
16	SGSVDYEEFLIGIAVCCRGT	94	0.80
16	ICPQGGISPVGSAHANAPPP	523	0.80
16	VQSTRVGAGGGAGGAGPANS	4	0.80
16	ASSSEGYGRSFDEESSGASS	292	0.80
16	GESVSDAAPVAGRGEVDLTR	1978	0.80
17	PAPPPIVPTSSGGVPAPGGV	547	0.79
17	GGGSRPVSVLPSRQSSEASV	503	0.79
17	SATPPVAAEAGSPGVSGALL	2032	0.79
17	NHGAPVATAGPPAALRPPVS	1847	0.79
17	LFSPQCQRTPQNGGSSGTAG	182	0.79
17	LLLRGRLPFPINQAFGHPSF	1724	0.79
17	ELVRGGELFDLIQQNHRLPE	1610	0.79
17	NELYAIKVIDKGKINGHERE	1556	0.79
17	AAIARAHDDPLACSGHSPRD	1286	0.79
17	EAVAVASVPGTPTTGATTAV	1060	0.79
18	PSKGPTKSAMLLQAEKDKTR	645	0.78
18	QEAPGTVSSPTGEQTGAPPA	353	0.78
18	APGCSDLSSASSGTQRRGTE	2077	0.78
18	ASPASLLNLTLQDGSEGRRM	2011	0.78
18	DEVRHSTRYGEERTMACCPE	1812	0.78
18	VHRDLKPENILLTDRTPNAT	1652	0.78
18	GGASGSADPSGGPGAEEDRV	1371	0.78
19	PSRQSSEASVICPQGGISPV	513	0.77
19	AAVSPSSLPAGSLDEVPESG	1954	0.77
19	YENTPVSFDGAVWREVSSSA	1744	0.77
19	ETLYIVMELVRGGELFDLIQ	1603	0.77
19	LLSEQVGGRQYGFAIVHPKG	1478	0.77
19	GASARAQLPYREGELRQADL	1266	0.77
20	PLPGLWGERLFQKFDFKGSG	76	0.76
20	PRAETQENETGLEELGEGAT	582	0.76
20	FGLSTLCAPNEVLHQPCGTL	1677	0.76
20	ATIKLTDFGLSTLCAPNEVL	1670	0.76
20	KHLHQWQARYYVLVDNMLYY	1437	0.76
20	CSGHSPRDLYSCPNCCNPLL	1298	0.76
20	AGGGAGGAGPANSLAFSTQL	11	0.76
21	GREGGSGKVFRRSKLLSSRT	778	0.75
21	VCVSDFVPSQQYVATGSGLS	243	0.75
21	AVSISSAPPAARTEGDTGPV	1932	0.75
21	KNIDVYISQLDEVRHSTRYG	1802	0.75
21	ELVAMLSNLPNLDRYMSIRK	137	0.75
21	REGELRQADLAAIARAHDDP	1276	0.75
21	VFDLNSDGYIQKSELVAMLS	124	0.75
21	TAPGTSVTHTATATAVQGPP	1135	0.75

**Table 4:** Conformational B-cell epitopes of CDPK7 protein predicted by the ElliPro server.

Residues	Number of residues	Score	3D structure
A:V1431, A:L1432, A:Y1433, A:K1434, A:K1435, A:G1436, A:K1437, A:H1438, A:L1439, A:H1440, A:Q1441, A:W1442, A:Q1443, A:A1444, A:R1445, A:Y1456, A:Y1457, A:R1458, A:R1459, A:K1460, A:G1461, A:D1462, A:A1463, A:K1464, A:P1465, A:R1466, A:G1467, A:F1468, A:E1477, A:L1478, A:L1479, A:S1480, A:E1481, A:Q1482, A:V1483, A:G1484, A:G1485, A:R1486, A:Q1487, A:Y1488, A:G1489, A:L1504, A:L1505, A:F1506, A:A1507, A:N1508, A:S1509, A:A1510, A:K1511, A:Q1513, A:R1514	51	0.82	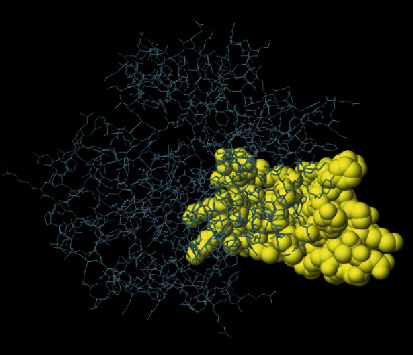
A:V1493, A:H1494, A:P1495, A:K1496, A:G1497, A:E1498, A:T1499, A:V1500, A:S1501, A:K1502, A:R1503	11	0.755	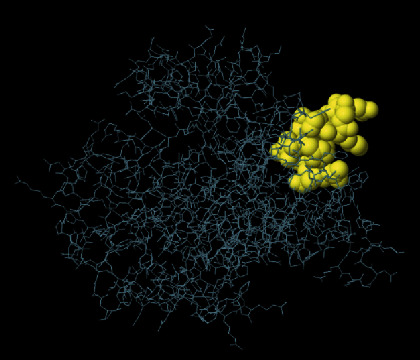
A:A1528, A:L1529, A:E1530, A:Q1531, A:L1532, A:Y1533, A:Q1534, A:V1535, A:G1536, A:E1537, A:Q1538, A:H1541, A:I1546, A:Y1548, A:K1549, A:G1550, A:I1551, A:H1552, A:R1553, A:A1554, A:T1555, A:N1556, A:E1557, A:L1558, A:L1611, A:V1612, A:R1613, A:G1614, A:Q1623, A:N1624, A:H1625, A:L1627, A:P1628, A:E1629, A:L1630, A:H1631, A:N1633, A:R1634, A:T1664, A:D1665, A:R1666, A:T1667, A:P1668, A:N1669, A:A1670, A:V1699, A:A1700, A:P1701, A:L1704, A:T1705, A:M1706, A:L1726, A:R1727, A:G1728, A:R1729, A:L1730, A:P1731, A:F1732, A:P1733, A:I1734, A:N1735, A:Q1736, A:A1737, A:F1738, A:G1739, A:P1741, A:S1742, A:F1743, A:Y1744, A:E1745, A:N1746, A:T1747, A:P1748, A:V1749, A:S1750, A:F1751, A:D1752, A:G1753, A:A1754, A:V1755, A:W1756, A:E1758, A:V1759, A:S1760, A:S1761, A:S1762, A:A1763, A:K1764, A:D1765, A:V1768, A:R1769, A:L1771, A:Q1772, A:P1773, A:N1774, A:P1775, A:R1776, A:R1777, A:R1778	99	0.677	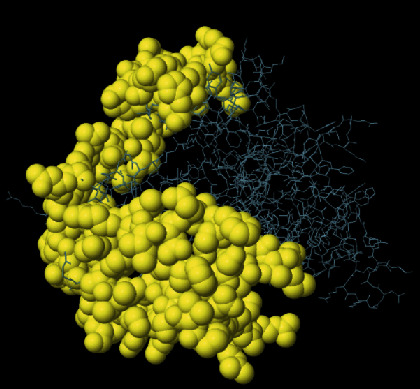
A:F1544, A:D1565, A:K1566, A:G1567, A:K1568, A:I1569, A:N1570, A:G1571, A:H1572, A:E1603, A:T1604, A:Y1606	12	0.645	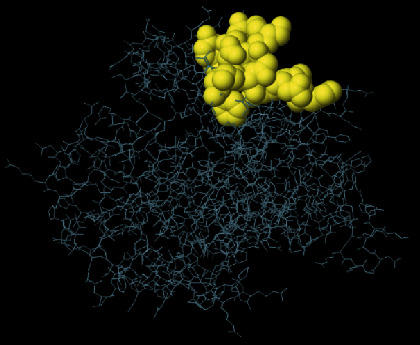
A:C1683, A:A1684, A:P1685, A:N1686, A:E1687, A:V1688, A:L1689, A:Q1691, A:P1692, A:C1693	10	0.535	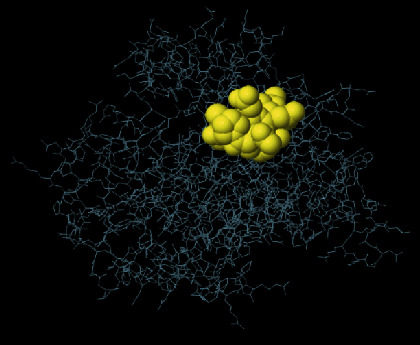

**Table 5:** IC_50_ values for CDPK7 binding to MHC class I molecules obtained using the IEDB^a^.

MHC-II allele^b^	Start-stop^c^	Peptide sequence	Percentile rank^d^	Antigenicity
	CDPK7		CDPK7	
H2-Db	1882-1891	SSPSSLPTPI	0.15	0.4079
	143-152	SNLPNLDRYM	0.21	-0.4284
	1637-1646	SQLLSTVYYL^∗^	0.24	0.9146

H2-Dd	647-656	KGPTKSAMLL^∗^	0.18	0.9201
	1612-1621	VRGGELFDLI	0.28	0.1770
	1483-1492	VGGRQYGFAI	0.64	0.1561

H2-Kb	1721-1730	IMYLLLRGRL^∗^	0.55	1.4751
	798-807	SSASFSSRGM^∗^	1.0	1.3623
	1643-1652	VYYLHKCGIV	1.2	0.1373

H2-Kd	1474-1483	CYVELLSEQV^∗^	0.79	0.5079
	1445-1454	RYYVLVDNML^∗^	1.15	0.9079
	822-831	GYSASGGMIV^∗^	1.3	0.9579

H2-Kk	1555-1564	TNELYAIKVI	0.12	0.2695
	1084-1093	SEGPATTPSI	0.75	0.2366
	694-703	DVEGIVDKII	1.5	0.1511

H2-Ld	1589-1598	HPNVIYMKEL	3.8	0.2773
	1729-1738	RLPFPINQAF	4.2	0.3317
	279-288	YEPHPLLARL	4.6	0.1027

^a^The immune epitope database (http://tools.iedb.org/mhci/). ^b^H2-Db, H2-Dd, H2-Kb, H2-Kd, H2-Kk, and H2-Ld alleles are mouse MHC class I molecules. ^c^Ten amino acids for analysis were used each time. ^d^Low percentile rank = high level binding; high percentile rank = low level binding; IC_50_ values = percentile rank. ∗ indicates potential antigenic epitopes (threshold = 0.5).

**Table 6:** IC_50_ values for CDPK7 binding to MHC class II molecules obtained using the IEDB^a^.

MHC-II allele^b^	Start-stop^c^	Peptide sequence	Percentile rank^d^	Antigenicity
	CDPK7		CDPK7	
H2-IAb	1109-1123	AAGAAAAAATAAAAA^∗^	0.07	0.8045
	1108-1122	AAAGAAAAAATAAAA^∗^	0.08	0.8354
	1110-1124	AGAAAAAATAAAAAF^∗^	0.08	0.7176

H2-IAd	1035-1049	SETQPAMASVASGSS^∗^	0.13	0.6766
	1034-1048	GSETQPAMASVASGS^∗^	0.15	0.7059
	1036-1050	ETQPAMASVASGSSP^∗^	0.25	0.6536

H2-IEd	1451-1465	DNMLYYYRRKGDAKP^∗^	0.14	0.6972
	1452-1466	NMLYYYRRKGDAKPR^∗^	0.14	0.8298
	1450-1464	VDNMLYYYRRKGDAK^∗^	0.19	0.6159

^a^The immune epitope database (http://tools.immuneepitope.org/mhcii). ^b^H2-IAb, H2-IAd, and H2-IEd alleles are mouse MHC class II molecules. ^c^Fifteen amino acids for analysis were used each time. ^d^Low percentile rank = high level binding; high percentile rank = low level binding; IC_50_ values = percentile rank. ∗ indicates potential antigenic epitopes (threshold = 0.5).

**Table 7:** Predicted CDPK7 epitopes by CTLpred^a^.

Peptide rank	Start position^b^	Sequence	Score (ANN/SVM)^c^	Antigenicity
1	280	EPHPLLARL	0.83/1.3591088	0.0131
2	1716	WSIGVIMYL	0.96/1.1120848	0.1711
3	1398	GSSRVFTRC	0.94/1.0685326	-0.7197
4	1187	ARDDDVYER	0.65/1.3441588	0.3493
5	715	SFPEFKTWL^∗^	0.98/0.95345497	1.0485
6	1763	AKDLIVRML^∗^	0.98/0.89030833	0.8096
7	724	ERNEGILSM^∗^	0.65/1.0757075	0.5393
8	470	ASRFTSAIK^∗^	0.80/0.85963689	1.0303
9	1573	ERELLRSEM^∗^	0.51/1.0720792	0.9337
10	1188	RDDDVYERI	0.85/0.73017891	0.0942
11	1666	RTPNATIKL	0.99/0.58481613	0.2323
12	32	KECLKQYLK^∗^	0.99/0.58376856	1.2628
13	1411	WELSKCAEM	0.19/1.3750392	0.3168
14	1749	VSFDGAVWR^∗^	0.96/0.59370426	1.2284
15	743	GLQGNALYR^∗^	0.99/0.54484483	1.4369

^a^CTLpred, available online at http://www.imtech.res.in/raghava/ctlpred/index.html. ^b^Nine amino acids for analysis were used. ^c^The default artificial neural network (ANN) and support vector machine (SVM) cut-off scores were set 0.51 and 0.36, respectively. ∗ indicates potential antigenic epitopes (threshold = 0.5).

## Data Availability

The datasets used and/or analysed during the current study are available from the corresponding author on reasonable request.

## Abbreviations

3D: Three-dimensional
ACC: Auto cross covariance
ANN: Artificial neural network
CD: Cluster of differentiation
CDPK: Calcium-dependent protein kinase
CTL: Cytotoxic T-lymphocyte
GOR: Garnier-Osguthorpe-Robson
GRAVY: Grand average of hydropathicity
IC_50_: Half-maximal inhibitory concentration
IEDB: Immune epitope database
IFN-*γ*: Interferon-*γ*
MHC: Major histocompatibility complex
MW: Molecular weight
PDB: Protein data bank
pI: Isoelectric point
PTM: Post-translational modification
SVM: Support vector machine
*T. gondii*: *Toxoplasma gondii*.
